# Ectopic Expression of a Maize Gene *ZmDUF1645* in Rice Increases Grain Length and Yield, but Reduces Drought Stress Tolerance

**DOI:** 10.3390/ijms24129794

**Published:** 2023-06-06

**Authors:** Yaqi Li, Wei Wang, Changqiong Hu, Songjin Yang, Chuan Ma, Jiacheng Wu, Yuwei Wang, Zhengjun Xu, Lihua Li, Zhengjian Huang, Jianqing Zhu, Xiaomei Jia, Xiaoying Ye, Zhiyuang Yang, Yongjian Sun, Huainian Liu, Rongjun Chen

**Affiliations:** 1 State Key Laboratory of Crop Gene Exploration and Utilization in Southwest China, Rice Research Institute of Sichuan Agricultural University, Chengdu 611130, China; 2 Demonstration Base for International Science & Technology Cooperation of Sichuan Province, Sichuan Agricultural University, Chengdu 611130, China; 3 Crop Ecophysiology and Cultivation Key Laboratory of Sichuan Province, Rice Research Institute of Sichuan Agricultural University, Chengdu 611130, China

**Keywords:** rice, maize, DUF1645 protein family, grain size, cytokinin

## Abstract

As the human population grows rapidly, food shortages will become an even greater problem; therefore, increasing crop yield has become a focus of rice breeding programs. The maize gene, *ZmDUF1645*, encoding a putative member of the DUF1645 protein family with an unknown function, was transformed into rice. Phenotypic analysis showed that enhanced *ZmDUF1645* expression significantly altered various traits in transgenic rice plants, including increased grain length, width, weight, and number per panicle, resulting in a significant increase in yield, but a decrease in rice tolerance to drought stress. qRT-PCR results showed that the expression of the related genes regulating meristem activity, such as *MPKA*, *CDKA*, a novel crop grain filling gene (*GIF1*), and *GS3*, was significantly changed in the *ZmDUF1645*-overexpression lines. Subcellular colocalization showed that *ZmDUF1645* was primarily localized on cell membrane systems. Based on these findings, we speculate that *ZmDUF1645*, like the *OsSGL* gene in the same protein family, may regulate grain size and affect yield through the cytokinin signaling pathway. This research provides further knowledge and understanding of the unknown functions of the DUF1645 protein family and may serve as a reference for biological breeding engineering to increase maize crop yield.

## 1. Introduction

A doubling of rice production per hectare is needed to meet the main calorific intake for half of the world’s population by 2050 [1,2,3]. Grain yield in rice is determined by three factors: number of panicles, number of grains per panicle, and grain weight [3,4]. Grain weight is mainly determined by grain shape and size, which are in turn determined by grain length, width, thickness, and filling degree [5]. Once the number of grains per panicle and panicles per plant reach optimal levels, an improvement in grain weight becomes important for further increasing the grain yield in breeding programs [6,7]. Of these traits, grain weight is the most important, as it is measured as a 1000-grain weight and is mainly restricted by the grain shape, and characterized by grain length, width, thickness, and filling degree [3,8]. These four parameters are positively correlated with the grain weight. Several genes affecting grain size have been cloned from rice varieties through quantitative trait locus (QTL) analysis, and to date, *GS3* [9,10], *qGL3/qGL3.1* [5], *GW2* [11], *GIF1* [12], *GS5* [13], and *GL7/GW7* [14,15] have been isolated as the genes that increase grain length. Among them, *GS3* and *qGL3/qGL3.1* are the main QTLs that regulate grain length by controlling the number of cells in the glumes [5,9,16]. *GW2*, which encodes a RING-type E3 ubiquitin ligase, negatively regulates grain width, weight, and yield by negatively regulating cell division in the shell [11,17]. *GS5* encodes a hypothetical serine carboxypeptidase that positively regulates the grain size by regulating the grain width, filling, and weight [12]. *GIF1* can positively control grain filling to improve crop yield or quality, or improve resistance or storage stability [12]. *OsSPL4* is a key regulator of grain size and provides a strategy for panicle architecture and grain size modification for yield improvement in rice [18].

Rice (*Oryza sativa*) is one of the most important food sources, but crop yield is controlled by multiple genes simultaneously and is heavily influenced by the environment [19,20,21]. Abiotic stresses such as drought, high/low temperatures, and salinity cause substantial yield losses annually [22,23]. Drought is a major environmental factor that adversely affects plant growth and limits agricultural productivity [24]; therefore, understanding the mechanistic effects of drought stress on rice will contribute to the better utilization of water resources in rice production [25]. The genes and their molecular functions determine the seed structure, components, and the quality of rice [26]; for example, the transcription factor AtNF-YB1 enables drought resistance in *Arabidopsis thaliana* [24]. Studies showed that OsGRP3 enhanced the drought resistance of rice [27], and the *AtEDT1/HDG11* gene enhanced the drought resistance and improved the yield of rice [28].

In eukaryotes, there are highly conserved unknown functional domain proteins (DUFs) that are part of several gene families encoding proteins of which the functions are not yet characterized [24]. Despite extensive research, there are still about 4000 poorly understood DUFs, accounting for more than 22% of all entries in the Pfam database [29]. Studies have shown that proteins containing DUF domains play important roles in plant growth and development, defense against diseases, and adaptive responses to stress. For example, *ESK1*, a member of the DUF231 domain protein in *Arabidopsis*, is a new negative regulator of cold stress [30], while *TBR* and *TBL3*, two other members, are involved in cellulose synthesis and secondary cell wall deposition [31]. The genes *G1*, *TH1/BSG1*, and *AFD1* containing DUF640 affect plant height, flower development, and yield by regulating cell division and expansion-related genes [32,33,34]. A novel gene encoding a conserved DUF581 domain has been shown to enhance salt stress tolerance in *Arabidopsis thalianass* [19,35].

Therefore, studying DUFs is of great significance for exploring various complex physiological and biochemical processes and their molecular mechanisms in plants. One member of the DUF1645 protein family in *Arabidopsis*, *AT1G23710*, has been shown to be induced by various environmental stress factors, including salt, cold, drought, ABA, and oxidative stress. Several studies have also demonstrated that *AT1G23710* expression is altered during processes such as cold acclimation, pollen germination, and pollen tube growth [24,36,37]. In this study, we cloned and identified the maize gene *ZmDUF1645*, which encodes the protein DUF1645 with an unknown function. We intend to make a preliminary analysis of the expression and function of this gene, so as to provide more resources for improving rice yield, quality and stress resistance.

## 2. Results

### 2.1. RNA-Seq Analysis of Gene Expression in Maize

**Figure 1:**
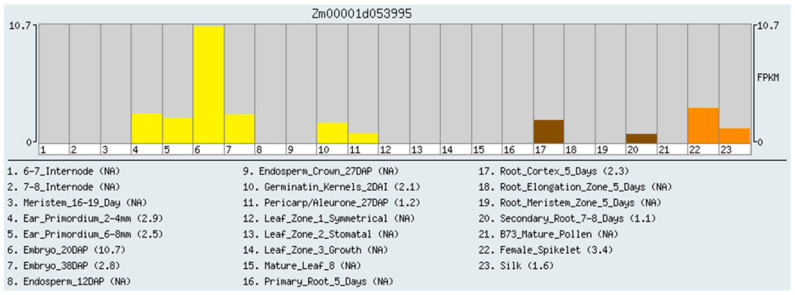
The expression level of ZmDUF1645 in different tissues of maize.

The reads were mapped to the B73 RefGen_v4 using STAR; the transcripts were detected from 43,781 genes. The complete dataset contains 1.35 billion uniquely mapped reads, with an average of 19.9 million uniquely mapped reads per biological replicate. These data show that the expression of this gene was the highest in maize embryos, followed by female spikelets. There was a significant expression level in the primordium and root, and the least expression in the secondary root (Figure 1).

### 2.2. Sequence and Phylogenetic Analysis of ZmDUF1645

**Figure 2:**
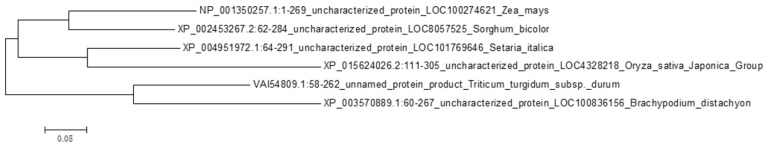
Phylogenetic tree analysis of ZmDUF1645.

We cloned the full-length cDNA of *ZmDUF1645* (Zm00001d053995), located on chromosome 4, from the maize-inbred line, B73. The gene has one exon and potentially encodes an ORF of 267 amino acids, with a predicted molecular mass of 27.51kDa and pI of 9.43. According to the predicted protein sequence of *ZmDUF1645* and analysis using BLAST on the NCBI website (http://www.ncbi.nlm.nih.gov/, accessed on 8 May 2020), the full-length protein sequence of *ZmDUF1645* was identified as being consistent with an uncharacterized protein (NP_001350257.1) encoded by LOC100274621 from maize B73. The BLAST analysis showed that *ZmDUF1645* shares some similarities with genes from other plant species (Figure 2). We constructed a phylogenetic tree by using the full-length amino acid sequence of the gene and the amino acid sequence of the gene encoding the same unknown functional protein DUF1645 in other species. The obtained dendrogram indicated that *ZmDUF1645* has a certain similarity with its ortholoys in *Sorghum bicolor*, *Setaria italica*, *Triticum turgidum subsp Durum*, *Brachypodium distachyon*, and the *Oryza sativa Japonica* Group (Figure 2). Moreover, among these species, it has been reported that the *Oryza sativa Japonica* Group (NCBI accession AK108331.1) and *ZmDUF1645* have the same effect on regulating grain length [23].

### 2.3. ZmDUF1645 Is Mainly Located in the Cell Membrane System

**Figure 3:**
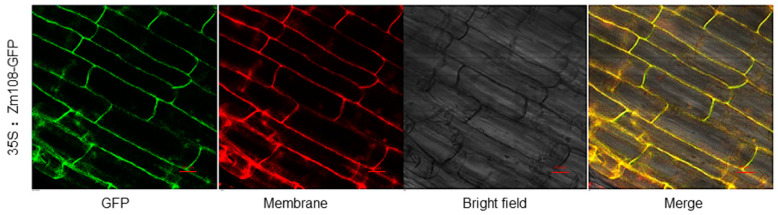
Subcellular localization of ZmDUF1645 in rice roots. Green represents the GFP signal of ZmDUF1645-GFP protein; red represents Nervered plasma membrane localization signal; Yellow indicates that the GFP signal of ZmDUF1645-GFP protein is consistent with the plasma membrane localization signal. Scale bars are 20 µm.

To examine the subcellular localization of the ZmDUF1645 protein in plant cells, we tested the 35S: *ZmDUF1645*-GFP transgenic rice roots. The transient expression of 35S: *ZmDUF1645*-GFP in rice root cells clearly shows that the GFP signal of the *ZmDUF1645*-GFP fusion protein is only observed in the membrane system, and is consistent with the Nervered plasma membrane localization signal (Figure 3). Therefore, it is preliminarily determined that *ZmDUF1645* is mainly located in the membrane system.

### 2.4. ZmDUF1645 Overexpression Significantly Changes Agronomic Traits in Rice

**Figure 4:**
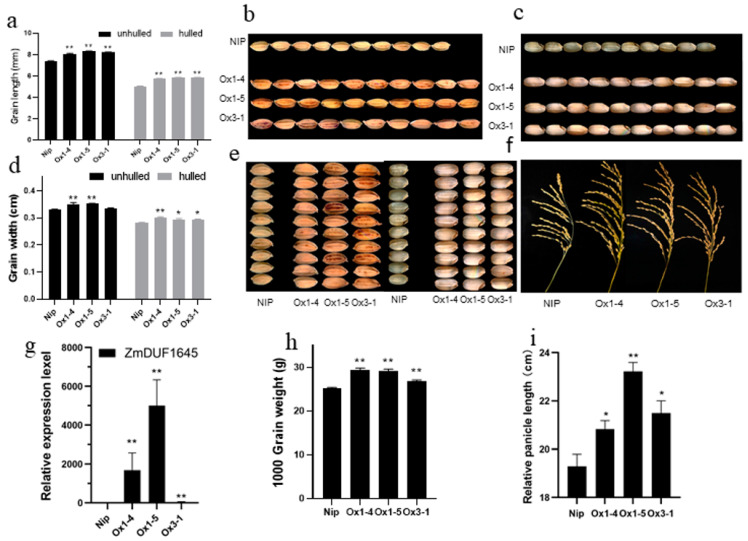
Comparison of grain size between ‘Nipponbare’ (NIP) and ZmDUF1645-OE. (**a**–**c**) Comparison of the grain length ((**a**), n = 10) of unhulled seeds and the grain length ((**b**), n = 10) of hulled seeds between NIP and ZmDUF1645-OE. (**d**,**e**) Comparison of the grain width ((**c**), n = 10) of unhulled seeds and the grain width ((**d**), n = 10) of hulled seeds between NIP and ZmDUF1645-OE. (**f**,**i**) Comparison of panicle phenotypes and panicle length between the NIP and OE lines. (**g**) The expression level of ZmDUF1645 in different tissues of overexpressing transgenic rice lines. (**h**) Comparison of 1000-grain weight (n = 3). Data are presented as the mean ± SD. Student’s *t*-tests were used to determine the *p*-values. * *p* ≤ 0.05, ** *p* ≤ 0.01. Scale bar, 1 cm for comparison of grain length and 0.2 mm for comparison of grain width.

**Figure 5:**
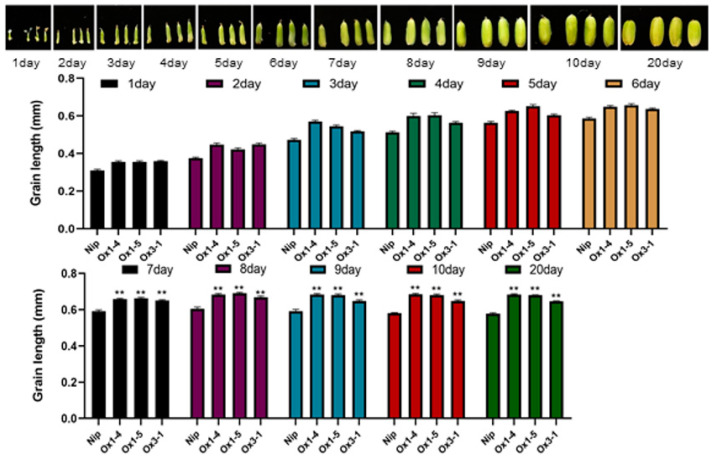
Grain length at different stages of grain filling (n = 10). Data are the mean ± SD. Student’s *t*-tests were used to generate the *p*-values. ** *p* ≤ 0.01.

The impact of this gene on the growth and development of rice was analyzed in the *ZmDUF1645*-overexpressed transgenic rice. A total of 26 transgenic plant lines (T3) were obtained, as confirmed by PCR and hygromycin screening analysis (Figure 4g). Three strains (OE1, OE3, and OE5) were selected for phenotypic analysis; the results showed that all transgenic lines had a generally normal phenotype during the vegetative growth stage. However, during the process of plant reproduction and development, changes in agronomic traits were observed under field conditions. Compared to the wild-type, the length of unshelled and shelled grains increased by 11.0% and 15.2%, respectively (Figure 4a–c). The width of the unshelled and shelled grains increased by 4.7% and 5.3%, respectively (Figure 4e), the weight of 1000 grains increased by 12.5% (Figure 4h), and the panicle length increased by 13.3% (Figure 4f,i). To analyze the effect of *ZmDUF1645* overexpression on grain development, the phenotype of the grains was observed at different stages of grain filling. The results showed that from the seventh day of observation, the grain length of the *ZmDUF1645*-OE plants was significantly longer than that of the wild-type plants (Figure 5).

### 2.5. ZmDUF1645 Affects Cytokinin Signaling

**Figure 6:**
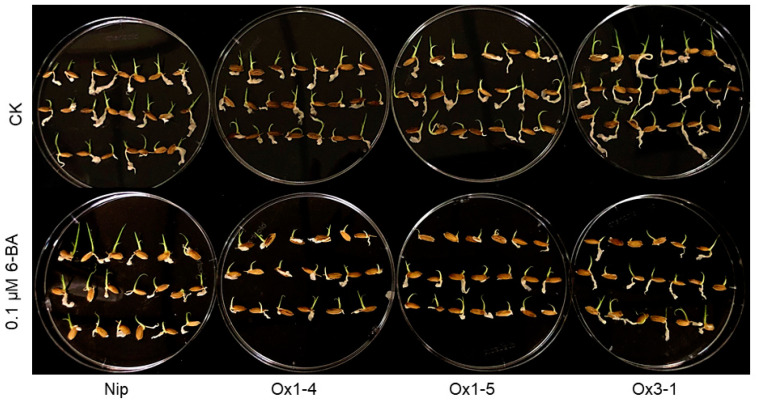
The response of ZmDUF1645 overexpression to exogenous hormone, 6-BA. Phenotypic differences of plants overexpressing ZmDUF1645 and the wild-type plants treated with 0 and 1 μM of 6-BA. n = 20 plants per treatment.

Based on the results of the quantitative analysis of cytokinin signaling genes and cyclin genes from this study and previous research, we conducted a 6-benzylaminopurine (6-BA) sensitivity test on the *ZmDUF1645*-overexpression line [38]. The results showed that after 6-BA treatment, the shoot elongation of the *ZmDUF1645*-overexpression line was significantly inhibited compared to the wild type (Figure 6). This suggests that the overexpression line is more sensitive to 6-BA and that *ZmDUF1645* is involved in the biosynthesis and signal transduction of cytokinins in rice.

### 2.6. ZmDUF1645 May Act through the Cytokinin Signal Transduction Pathway, like OsSGL

**Figure 7:**
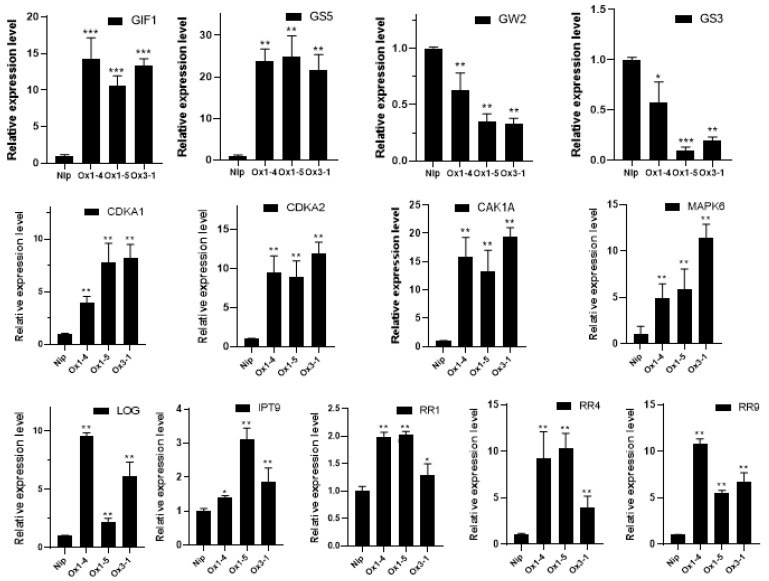
Relative expression levels of the analyzed genes in three ZmDUF1645-overexpressing transgenic lines and the Nip. Genes were grouped based on their function and family. Error bars indicate ± SE (n = 3). Asterisks indicate significant differences between the transgenic lines and the wild type (* *p* < 0.05, ** *p* < 0.01, *** *p* < 0.001).

To gain further insight into the function of *ZmDUF1645*, we selected 28 target genes based on the research results of *OsSGL*, a hypothetical member of the DUF1645 protein family with an unknown function [23]. We then analyzed their expression levels in the three overexpressed lines (OE1, OE3, and OE5) and the wild-type line using qRT-PCR. The selected genes included 7 grain-regulating genes, 12 cytokinin signaling-related genes, and 9 cell cycle regulation-related genes (Supplementary Table S1). Compared to the wild type, the transcription levels of the grain size-positive regulating genes, *GIF1* and *GS5* (Figure 7), were significantly increased in the overexpressed lines, while the expression of the grain size negative-regulating genes, *GS3* and *GW2*, was significantly reduced (Figure 7). The expression levels of the putative G1/S-phase genes *CAK1*, *CDKA1*, and *CDKA2* also increased in the transgenic plants (Figure 7). The expression levels of six genes related to the cytokinin signaling pathway, *MAPK*, *LOC*, and *IPT9*, and three A-type OsRR (response regulator) genes (*RR1*, *RR4*, and *RR9*) showed significant changes (Figure 7). The expression levels of the other genes analyzed did not show significant differences between the transgenic and wild-type samples. The correlation between changes in the gene transcription levels and *ZmDUF1645* overexpression suggests that *ZmDUF1645* may play an important role in regulating meristem activity.

### 2.7. ZmDUF1645 Overexpression Decreases Tolerance to Drought Stress

**Figure 8:**
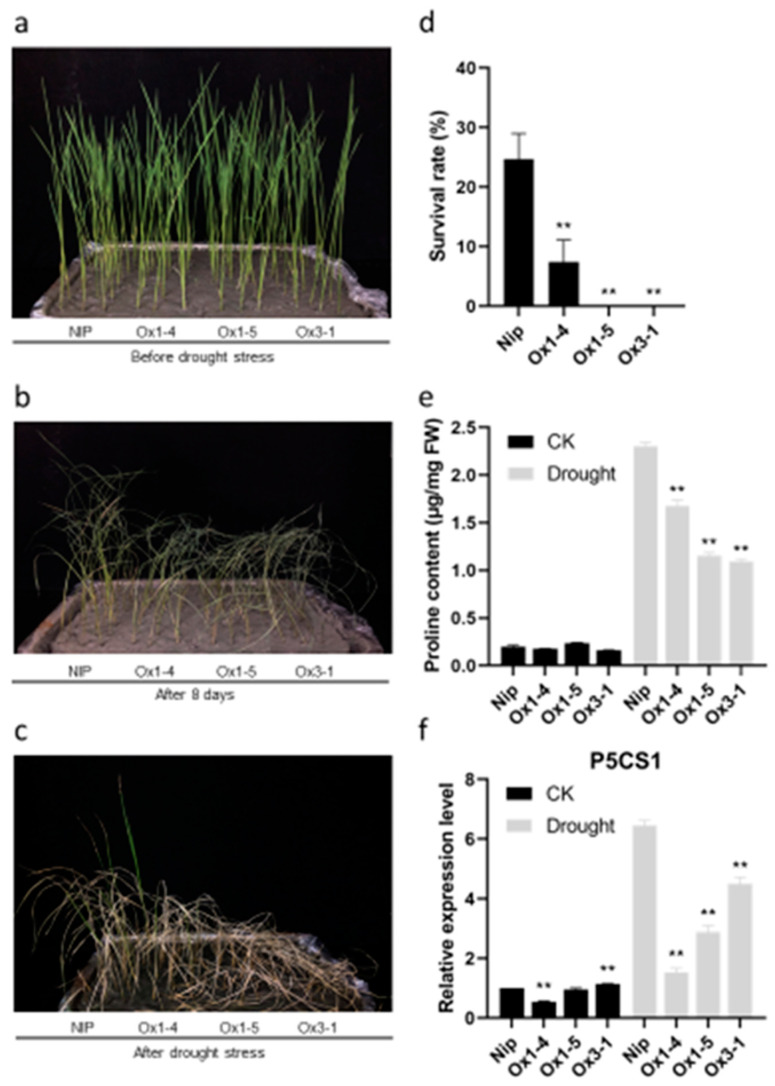
*ZmDUF1645* reduces tolerance to drought stress. (**a**–**d**) Comparison of the relative survival rate of 21-day-old seedlings after 8 days of drought treatment and 7 days of recovery (n = 27). (**e**) Comparison of the proline content in 21-day-old seedlings after 5 days, with or without drought treatment. (**f**) Comparison of the relative expression level of *P5CS1* in 21-day-old seedlings after 5 days, with or without drought treatment. Error bars represent ± SE (n = 3). Asterisks indicate significant differences between the transgenic lines and wild type (** *p* < 0.01).

The effect of abiotic stress on the growth, development, and yield of crops is significant, so we studied the response of *ZmDUF1645* transgenic seedlings to abiotic stress. As shown in Figure 8a–d, the survival rate of the *ZmDUF1645*-overexpression lines was significantly lower than that of the wild type after drought treatment. In order to understand the possible reasons for the decrease in drought resistance of ZmDUF1645, we measured the proline content and the expression of the proline biosynthesis gene, *OsP5CS1* (LOC_Os05g38150), in wild-type and *ZmDUF1645* transgenic plants, under both drought and normal conditions. As shown in Figure 8e,f, under normal conditions, there was little difference in the proline content and *OsP5CS1* expression between the wild-type and *ZmDUF1645*-overexpression lines. However, after drought treatment, the proline content and the expression of *OsP5CS1* in the *ZmDUF1645*-overexpression lines were significantly lower than those in the wild type. These results suggest that *ZmDUF1645* may decrease the drought tolerance of rice by reducing the proline content.

### 2.8. ZmDuF1645 Increases the Number of Stomata and the Degree of Stomatal Opening and Closing

**Figure 9:**
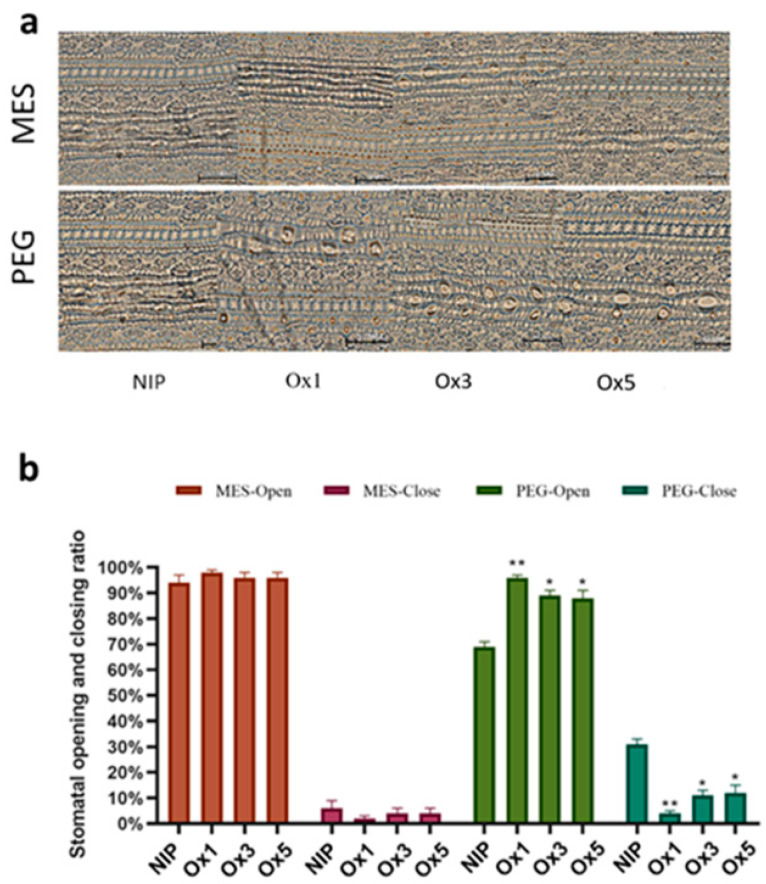
Increased stomatal number and opening and closing degree in *ZmDuF1645*-overexpression lines. (**a**) Comparison of stomatal number in rice leaves between the *ZmDuF1645*-overexpression lines and Nipponbare under a 10 × 20-fold microscope. (**b**) Comparison of stomatal opening and closing rate in rice leaves before and after treatment with the PEG solution. Error bars represent ± SE (n = 3). Significant differences between the transgenic lines and Nipponbare are indicated by asterisks (* *p* < 0.05, ** *p* < 0.01).

The stomata of plants are the main sites of transpiration, and the water loss of plants is closely related to the opening and closing degree of stomata. As shown in Figure 9a, the number of stomata in Nipponbare was significantly lower than that in the *ZmDUF1645*-overexpression lines in the same field of view under the 10 × 20-fold microscope. The observation showed that there was no difference in the stomatal opening and closing between Nipponbare and *ZmDUF1645*-overexpression lines before PEG treatment. However, after PEG treatment, the stomatal opening and closing degree of Nipponbare decreased significantly, while that of *ZmDUF1645*-overexpression lines remained high (Figure 9b). We speculate that the reduced drought resistance of the *ZmDUF1645*-overexpression lines may be related to its higher number of stomata and higher degree of opening and closing, thus resulting in its greater susceptibility to water loss.

## 3. Discussion

The gene *ZmDUF1645* is an intron-less gene that encodes a maize B73 uncharacterized protein and belongs to the DUF1645 protein family with unknown functions. In this study, it was found that overexpressing *ZmDUF1645* in rice plants positively affects its agronomic traits, including rice grain length, grain width, and 1000-grain weight, which may result in increased yield [39]. Another rice gene, *OsSGL*, has been studied and also belongs to the DUF1645 protein family [23,24]. Studies have shown that overexpressing *OsSGL* not only enhances agronomic traits and improves grain yield, but also improves the drought tolerance of rice [23,24]. However, it remains unclear whether the integrity of the DUF1645 domain is necessary for *OsSGL* to improve grain length and drought tolerance [23,24]. Some studies have shown that overexpressing *OsSGL* increases drought tolerance in rice and *Arabidopsis*, but overexpressing *ZmDUF1645* does not [23,24]. It is speculated that *ZmDUF1645*, as a foreign gene, can only regulate rice grain type and yield through the cytokinin (CK) signaling pathway, while *OsSGL*, as an endogenous gene, can participate in multiple unknown complex signal pathways to make rice resistant to drought. Therefore, it is believed that the DUF1645 domain regulates plant growth and yield, but its ability to regulate drought tolerance is different from *OsSGL*.

Cytokinins are synthesized by lonely guy (LOG), cytochrome P450 monooxygenase (CYP735As), and adenosine phosphate isopentenyl transferase (IPT), and regulate various biological processes through a complex signal network [40,41,42], including cell proliferation [43], reproductive development [44], seed yield [45], and response to environmental stress [46]. In the signaling pathway, type-A response regulators (RRs) play a crucial role in transmitting the signal, and most RRs are induced by cytokinins and are therefore useful markers to evaluate the cytokinin response [47,48]. Our results showed that the expression levels of the genes related to grain type regulation, such as *GS3*, *GS5*, cytokinin signal genes (RRs), *LOCs*, and cell cycle regulation genes *CDKA1*, and *CDKA2*, changed significantly in the *ZmDUF1645*-overexpression line, similar to the study on *OsSGL* regulation of grain size. Additionally, the results of the 6-benzylaminopurine (6-BA) sensitivity test, where the *ZmDUF1645*-overexpression line was more sensitive to 6-BA treatment, further support the role of *ZmDUF1645* in regulating cytokinin signaling. Therefore, we speculate that the DUF1645 protein family may regulate crop grain types through cytokinin signal transduction pathways [48].

In addition, in order to explore whether *ZmDUF1645*, like *OsSGL*, not only improves the grain yield, but also improves the drought tolerance of rice, we treated the 21-day-old rice seedlings with drought stress. The results showed that the survival rate of *ZmDUF1645*-OE lines was significantly lower than that of the wild type after drought treatment (Figure 8a–d). Proline is reported to be a major compatible solute that can protect subcellular structures and biomacromolecules from drought, heavy metals, salt and other stresses [49]. Proline is mainly synthesized from glutamate in plants, the first step of which is catalyzed by δ′-pyrroline-5-carboxylate synthetase (P5CS), which is rate-limiting [49,50]. Two *P5CS* gene homologues, *OsP5CS1* and *OsP5CS2*, have been reported in rice, in which the transcripts of *OsP5CS1* are up-regulated by NaCl, osmotic, dehydration, and the cold [51,52]. Therefore, we measured the proline content and the expression level of *OsP5CS1* in all lines before and after drought treatment. We can find that after drought treatment, the proline content and the expression of *OsP5CS1* in the *ZmDUF1645*-overexpression lines were significantly lower than those in the wild type (Figure 8e,f). Therefore, we speculate that *ZmDUF1645* reduces the tolerance of rice to drought stress by reducing proline biosynthesis. Many research studies have been carried out on heterologous expression. For example, the heterologous expression of the antifungal protein Ag-AFP from the fungal and the barley seed-specific chitinase II gene in wheat resulted in an enhanced resistance against mildew and rust [53]. The ectopic overexpression of *AcWRKY31* in pineapple, rice, and *Arabidopsis* resulted in plants that were excessively sensitive to drought and salt stress [54]. The heterologous expression of *ZmDUF1645* in rice may be the reason why its drought tolerance is different from *OsSGL*. On the other hand, combined with subcellular localization results, *ZmDUF1645* is localized in the cell membrane (Figure 3) rather than *OsSGL*, which is a nucleus-localized protein [19,24]. The ectopic expression of the transcription factor genes may be another reason the two genes differ in drought resistance [55].

## 4. Materials and Methods

### 4.1. Plant Materials and Growth Conditions

The seeds of the Nipponbare (NIP) rice cultivar wild-type (WT) and T3 transgenic plants were soaked in sterilized 3% H_2_O_2_ for 2 days, then washed three times under running water. The seeds were germinated in a liquid culture medium at 37 °C for 2–3 days, and then transferred to an incubator with a photoperiod of 12 h of light (30 °C)/12 h of dark (25 °C) for 14 days prior to RNA extraction. Some of the germinated seeds were cultured for 5 days before being transferred to the Greenhouse of Rice Research Institute and grown under natural conditions. Young panicles, 5–8 cm long, were harvested for samples at the booting stage, and the panicles, after flowering 2–5 days, were harvested for samples at the heading stage.

### 4.2. Phenotypic Measurements and Statistical Analysis

The harvested rice grains were air-dried and stored at room temperature for at least two months before testing. The fully developed grains (with and without hulls) were measured for grain length, width, and weight. Ten full seeds were randomly selected from each line and used to measure seed length and width by averaging the three measurements using a Vernier caliper. The weight of 100 seeds from each plant was measured to determine the 1000-seed weight. All experiments were performed at least three times using independent samples, and the data were analyzed using the SPSS 11.0 software. Student’s *t*-test was used to compare the means, and a *p*-value of less than 0.05 was considered statistically significant.

### 4.3. RNA Extraction, Synthesis, and Purification of cDNA

The RNA extraction procedure was performed as described previously [7]. The leaves were collected from the experimental and control groups, cut into pieces, and stored at −70 °C until needed. Total RNA was extracted from the frozen samples using TRIzol (Invitrogen, Burlington, ON, Canada) [56,57]. Reverse transcription was performed using HiScript^®^Ⅱ Q RT SuperMix for qPCR (+GDNA wiper, Vazyme, Beijing, China), and the cDNA was stored at −20 °C.

### 4.4. Quantitative Real-Time Polymerase Chain Reaction (qRT-PCR)

Three biological replicates were used for real-time qRT-PCR (Bio-Rad Powerpac300, Concord, CA, USA). The primers for the target gene (*ZmDUF1645*) were designed using the Primer Express 3.0 software, and the primers were tested via conventional RT-PCR and agarose gel electrophoresis. The qRT-PCR was performed using an ABI7900 and TB Green^®^ Premix Ex TaqTM Ⅱ (Tli RNaseH Plus). Each reaction mixture (10 mL) contained 2× Master Mix (5 mL), 0.3 mL of each primer (10 mmol/L), 1 mL of template RNA sample (40 ng), and 3.4 mL of RNase-free water. The thermal cycling program was as follows: initial denaturation for 5 min at 95 °C and 40 amplification cycles (15 s at 95 °C, 40 s at 58 °C, and 20 s at 72 °C). The raw data of RT-PCR were obtained by Bio-Rad CFX Manager, and the relative expression levels were calculated using the 2^−Ct^ method [58]. Ubiquitin 5 (*LOC_Os01g22490*) was used as the internal reference gene in this experiment.

### 4.5. Gene Cloning, Transgenic Constructions, Multiple-Sequence Alignment Analysis, Phylogenetic Analysis, and RNA-Seq Analysis of Gene Expression in Maize

In this study, the sequences for PCR were obtained from Gramene Basic Local Alignment Search Tool (BLAST) and the National Center for Biotechnology Information (NCBI) (https://www.ncbi.nlm.nih.gov/, accessed on 8 May 2020) search database. Specific primers were designed using the Primer Premier 5.0 software. The full-length or truncated coding sequences (CDS) of *ZmDUF1645* were amplified from the maize variety B73, and cloned into the multi-cloning sites of the binary expression vector D-163+1300; *ZmDUF1645* under the control of the CaMV35S promoter to create overexpression constructs that carried hptII (encoding hygromycin resistance) as a selection marker, and the restriction sites were HindIII and BamHI. The plasmid constructs were introduced into the Agrobacterium tumefaciens strain EHA105 and then transferred into the japonica variety Nipponbare. Multiple-sequence alignment analysis was performed using the DNAMAN 7.0 software. A phylogenetic tree was constructed using the MEGA 5.0 software based on the neighbor-joining method, as described previously [59]. The expression level of ZmDUF1645 in various tissues of maize was obtained from the maizeGDB database (https://www.maizegdb.org/, accessed on 8 May 2020). The RNA-seq gene atlas of maize-inbred B73 includes 68 different samples from 23 tissues with 3 biological replicates per tissue (2 replicates for vegetative meristem).

### 4.6. Subcellular Localization of the ZmDUF1645 Protein in Rice Roots

The full-length amino acid sequence of *ZmDUF1645* was fused with the GFP protein in the transient expression vector pAcGFP, under the control of the 35S promoter and the vector 35S; *ZmDUF1645*-GFP was transformed into rice callus via a Agrobacterium-mediated method. The callus that had differentiated into seedlings was then transferred to the field for planting until the seeds were harvested. Before the heading stage of the rice plants, the leaves were cut for a PCR-positive identification to collect the positive seeds. The harvested positive seeds were germinated in the dark in a hydroponic solution containing 50 mg/L hygromycin for 5–7 days, and the seedling roots were selected for filming. Nervered C2 (Fluorescent membrane dye) was added dropwise as a plasma membrane dye during filming and observed with a laser confocal microscope (Nikon A1 i90, LSCM, Tokyo, Japan) after a few minutes, under a 40 × 10 confocal microscope.

### 4.7. Abiotic Stress Treatment and Proline Content Determination

Sterilized seeds were cultured in a liquid medium at 37 °C for 2–3 days to germinate. The germinated seeds were transferred to pots in an incubator with a photoperiod of 12 h light (30 °C)/12 h dark (25 °C) for soil culture. The 21-day-old seedlings were treated without water for 8 days to observe their phenotype and the survival rate of all plants was counted after seven days of resuming watering. The experiment was repeated three times with 27 plants counted for each line.

As previously mentioned, when the seedlings were 21 days old and treated without water for 5 days, the aboveground parts of some seedlings were collected to extract RNA for qRT-PCR analysis, and the other part was used to measure the proline content. The PCR primers are listed in the Supplementary Table S1. The proline content was measured using a spectrophotometer, as described in [60].

### 4.8. Observation of Stomatal Number and Degree of Stomatal Opening and Closing in Leaves

Rice seedlings at an age of 4 to 6 weeks were used for this observation. The tips of the leaves were cut off and the middle and upper parts of the leaves at the same location were selected. The leaves were placed in an MES buffer (pH 6.1) and 20% PEG solution, respectively, and exposed to sunlight for 2 h. After removing the leaves, the surface liquid was dried, and a layer of colorless nail polish was applied. Once the nail polish was air-dried, it was covered with a layer of transparent tape, smoothed, torn off, and then stuck on a cover glass. Finally, the samples were observed under a low-power microscope.

## 5. Conclusions

The *ZmDUF1645* and rice *OsSGL* genes belong to the same DUF1645 protein family and are both involved in regulating grain types in crops through the cytokinin signal transduction pathway. Our speculation is that the DUF1645 domain has the function of regulating plant growth and yield, but further research is needed to determine its effect on plant drought tolerance. This study highlights the potential of using DUF1645 protein family genes as candidates for the molecular breeding of high-yield crops.

## Data Availability

All the data are illustrated in the figures and the Supplementary Data. All experiments of plants and field experiments were performed at our affiliated university. The datasets used and/or analyzed during the current study are available from the corresponding author on reasonable request.

## Supplementary Materials

The following supporting information can be downloaded at: https://www.mdpi.com/article/10.3390/ijms24129794/s1.
