# Supplementary material for: Ectopic Expression of a Maize Gene ZmDUF1645 in Rice Increases Grain Length and Yield, but Reduces Drought Stress Tolerance
Source: Int J Mol Sci. 2023 Jun 6;24(12):9794. doi: 10.3390/ijms24129794 (PMC10298434; doi:10.3390/ijms24129794)
Supplement: Supplementary file 1 [file ijms-24-09794-s001.zip › ijms-2415714-supplementary.pdf]

Table S1. PCR Primers.

Primer Name	Primer Sequences
ZmDUF1645	F:5'-ATGCGGGCACGAAGAATAAG-3'
	R:5'-TTGCCGTAGAAGAGCCTGTG-3'
GW2	F:5'-CTGCAGCAGGGAAGTGGT-3'
	R:5'-ATGTCTGAGCCCGCGTAG-3'
GS3	F:5'-CATCGGAGAAGCGAAGTCA-3'
	R:5'-CAGCAGCAGATCCAGGAGA-3'
GIF1	F:5'-CATCGCGCAACCCGAACATG-3'
	R:5'-TGTCGATCAGGCTCCTCAGAG-3'
GS5	F:5'-AGTGGACTGCTTCCAGGGAAG-3'
	R:5'-CACGCAGTACCGAGAACTGA-3'
RR1	F:5'-AGGATCAGCAGATGCATGAATG-3'
	R:5'-GAGACGCTGTACGTCCTTGCTT-3'
RR4	F:5'-TCTTCTGAGAATGTGCCTGCAA-3'
	R:5'-GCTTGACAGGTTTCAGGAAGAACT-3'
RR9	F:5'-TCATGAGGACAGCCCAATTTCTA-3'
	R:5'-TGCAGTAGTCTGTGATGATCAGGTT-3'
MAPK6	F:5'-ACAGAGCAGCCGAATTTTGAGA-3'
	R:5'-TTCAGCGAAGCTCACACTTGG-3'
IPT9	F:5'-GCTCTTGTTAGCCCATTCCTCTT-3'
	R:5'-TGGTTGTTCGGGTGTATCCTTT-3'
LOG	F:5'-AGCGAAGGATTCATAGCGGA-3'
	R:5'-CGGCATCTGATCGTCCCAA-3'
CDKA1	F:5'-GGTTTGGACCTTCTCTCTAAAATGC-3'
	R:5'-AGAGCCTGTCTAGCTGTGATCCTT-3'
CDKA2	F:5'-GGTTTGGACCTTCTCTCTAAAATGC-3'
	R:5'-AGAGCCTGTCTAGCTGTGATCCTT-3'
CAK1A	F:5'-GGTTTGGACCTTCTCTCTAAAATGC-3'
	R:5'-AGAGCCTGTCTAGCTGTGATCCTT-3'
P5CS1	F:5'-GCTGACATGGATATGGCAAAAC-3'
	R:5'-GTAAGGTCTCCATTGCATTGCA-3'
